# ALDH1 immunoexpression in epithelial and stromal cells of oral lichen planus and lesions with lichenoid inflammatory infiltrate

**DOI:** 10.4317/medoral.25861

**Published:** 2023-10-12

**Authors:** Michelle Danielle Porto Matias, Daniela Pereira Meirelles, Martinho Campolina Rebello Horta, Karine Duarte da Silva, Patrícia Carlos Caldeira, Maria Cássia Ferreira de Aguiar

**Affiliations:** 1 MSc Student. Department of Oral Pathology and Surgery, School of Dentistry, Universidade Federal de Minas Gerais, Belo Horizonte, Minas Gerais, Brazil; 2 PhD Student. Department of Oral Pathology and Surgery, School of Dentistry, Universidade Federal de Minas Gerais, Belo Horizonte, Minas Gerais, Brazil; 3 PhD Professor. Department of Dentistry, School of Dentistry, Pontifícia Universidade Católica, Belo Horizonte, Minas Gerais, Brazil; 4 PhD. Department of Oral Pathology and Surgery, School of Dentistry, Universidade Federal de Minas Gerais, Belo Horizonte, Minas Gerais, Brazil; 5 Professor. Department of Oral Pathology and Surgery, School of Dentistry, Universidade Federal de Minas Gerais, Belo Horizonte, Minas Gerais, Brazil; 6 PhD Professor. Department of Oral Pathology and Surgery, School of Dentistry, Universidade Federal de Minas Gerais, Belo Horizonte, Minas Gerais, Brazil

## Abstract

**Background:**

Oral Lichen Planus is a potential malignant disorder and shares clinical and histopathological features with other similar lesions. ALDH1 is a specific biomarker for stem cells identification, however its role in stromal cells of immune inflammatory infiltrate has not been explored. The aim of this study was to investigate the ALDH1 immunoexpression in epithelial and stromal cells of Oral Lichen Planus and other lesions with lichenoid inflammatory infiltrate.

**Material and Methods:**

64 samples of Oral Lichen Planus, Oral Lichenoid Lesions, Oral Leukoplakia and Unspecific Chronic Inflammation were included. ALDH1 was evaluated in both epithelium and stromal cells. ALDH1+ cells ≥ 5% were considered positive in epithelium. Stromal cells were evaluated semi quantitatively. Fields were ranked in scores, according to criteria: 1 (0 to 10%); 2 (11 to 50%) and 3 (>50%). The mean value of the sum of the fields was the final score. Statistical differences among groups were investigated, considering *p* < 0.05.

**Results:**

ALDH1 expression in epithelium was low in all groups without difference among them. ALDH1+ cells in the lamina propria were higher for Lichen Planus [2.0], followed by Leukoplakia [1.3], Lichenoid lesions [1.2] and control [1.1] (*p*<0.05).

**Conclusions:**

ALDH1 immunoexpression in epithelium of lichenoid potential malignant disorders did not show a contributory tool, however ALDH1 in stromal cells of lichen planus might be involved in the complex process of immune regulation associated with the pathogenesis of this disease.

** Key words:**Aldehyde dehydrogenase 1 family, oral lichen planus, leukoplakia, lichenoid lesions, immunohistochemistry.

## Introduction

Oral Potentially Malignant Disorders (OPMD) comprise a large and diverse group of mucosal disorders that can precede the diagnosis of oral squamous cell carcinoma. Different OPMD carry on variable malignant transformation rates and identifying which individual will develop cancer can be a challenge (1).

The exact mechanisms by which Oral Lichen Planus (OLP) and Oral Lichenoid Lesions (OLL) undergoes malignant transformation have not yet been determined, it is though that chronic immune stimulation and inflammation can cause growth signal dysregulation and this combines with oxidative stress to induce DNA damage (2,3).

Many biological markers have been employed to elucidate the malignant potential of OLP (4,5). ALDH1 is an isoform of dehydrogenase aldehyde expressed in humans as a cytosolic detoxifying isozyme. ALDH1 oxidizes retinol to retinoic acid, which, in turn, promotes the transcription of genes that participate in cell differentiation,

apoptosis, or even tumor growth being a specific biomarker for stem cells’ identification (6,7). In addition, retinoic acid has an important role in the control of immune inflammatory diseases (8,9). The immunohistochemical evaluation of ALDH1 in OPMD has been restricted to epithelium acting as a marker of cancer stem cell (10), meanwhile its potential in the inflammatory infiltrate has not been investigated. Further, the role of ALDH1 has not been explored in OLP and other lichenoid lesions. This study aimed to investigate ALDH1 expression in the epithelial and stromal cells of OLP, OLL and Leukoplakia (OLK). This study is an attempt to unravel the biology behind ALDH1 expression in epithelial and stromal cells of OLP and other lichenoid lesions.

## Material and Methods

- Case selection and sample

This cross-sectional study followed the Strengthening the Reporting of Observational Studies in Epidemiology (STROBE) guidelines. Cases with a histopathological diagnosis of OLP, OLL, and OLK were retrieved from the files of the Oral Maxillofacial Pathology Service (UFMG- Brazil) from 2011-2021. The elegibility criteria are presented in Table 1.

**Table 1:**
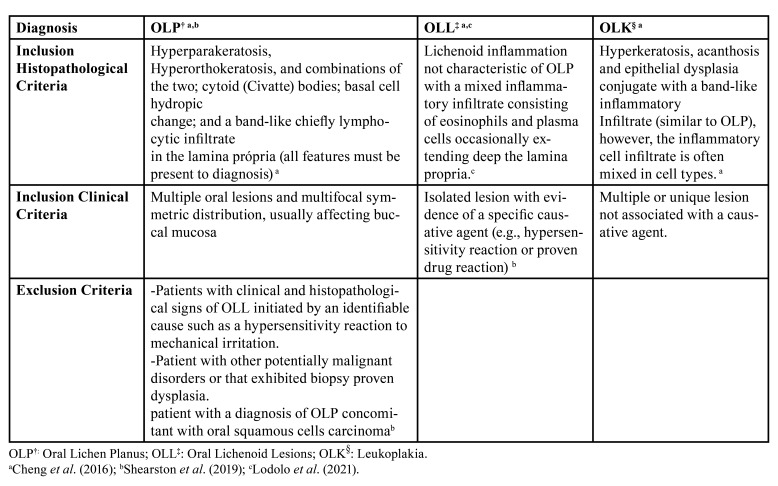
Eligibility criteria of study.

All cases were independently reviewed by three oral pathologists). Only cases filling the clinical and histopathological criteria (11-13) and having a concordant diagnosis of at least two examiners were included. OLK cases included histopathologically identified moderate or severe dysplasia.

Were selected for the study 16 OLP, 16 OLL, and 16 OLK, totalizing 48 cases. Sixteen cases diagnosed as Unspecific Chronic Inflammation (UCI) were included as controls. Those cases were nodules, ulcers, erosions featured by a mixed diffuse inflammatory infiltrate extending deeply into the lamina propria, without other specific diagnosis. Cases histologically diagnosed as lichenoid mucositis were excluded.

- Immunohistochemistry

Immunohistochemistry was performed on 4-μm thick sections, following standard protocols on silanized slides. The antigen retrieval was performed with TRILOGY™ Concentrate (Cell Marque, Rocklin, CA, US; 1:100) in pressure cooker 96°C for 30 min. Monoclonal anti-ALDH1 was used (clone 44 ALDH; Mouse; BD Bioscience, California, CA, US; 1:00). Next, the sections were incubated with a ready-to-use polymer (CRF Anti-Polyvalent HRP Polymer, ScyTek Laboratories, Logan, UT, USA, code ABZ125). 3.3’-Diaminobenzidine was used as the chromogen (Dako, Carpinteria, CA, US) and counterstained with hematoxylin.

- Immunohistochemical assessment

A two-step immunohistochemical analysis was set: a morphological and a quantitative, of the epithelium and stromal cells. For epithelium and stroma, cells exhibiting a brown staining in cytoplasm and cytoplasmatic membrane were considered positive, regardless the intensity.

All slides were scanned in low power (100x) to determine the positive areas. Then, the ALDH1+ areas were evaluated in a higher magnification (400x) to determine the staining pattern (confluents and isolated cells for epithelium) and the cell morphology (spindle or round for stromal cells).

A detailed descriptive analysis was jointly performed by three examiners at a Penta Head Light Microscope (Axioskop 2 Plus, Carl Zeiss). A semiquantitative analysis was made considering epithelium and stromal cells. All epithelium extension was evaluated, and epithelium was considered positive when 5% or more cells were stained at a hotspot area in a 200x magnification. Positive cases were classified as isolated or confluent ALDH1 in a 400x magnification (6,7).

The stromal cells evaluation was made in semiquantitative form according to Bednarz *et al*. (2015) (14), however the staining intensity was not considered. All fields at a horizontal axis, immediately bellow epithelium in the lichenoid infiltrate where positive cells were concentrated, were evaluated at high power (400x) by two jointly examiners. Fields were ranked in scores, depending on the percentage of positive cells, according to criteria: 1 (0 to 10%); 2 (11 to 50%) and 3 (more than 51%). The final score of each case was the mean value of the sum of the fields.

- Statistical Analysis

Statistical analysis was performed using the software SPSS - Statistical Package for the Social Sciences® (IBM - version 26.0, 2019, Chicago, IL, USA).

The epidemiological and clinical-pathological variables were analyzed descriptively. The Kolmogorov-Smirnov test was applied to verify the normality distribution of ALDH1 expression in the sample. Kruskal-Wallis test was applied to compare ALDH1 immunoexpression among groups, and the significance levels were adjusted with Bonferroni correction. Statistical significance was set as *p* < 0.05.

## Results

- Sample selection

**Figure 1:**
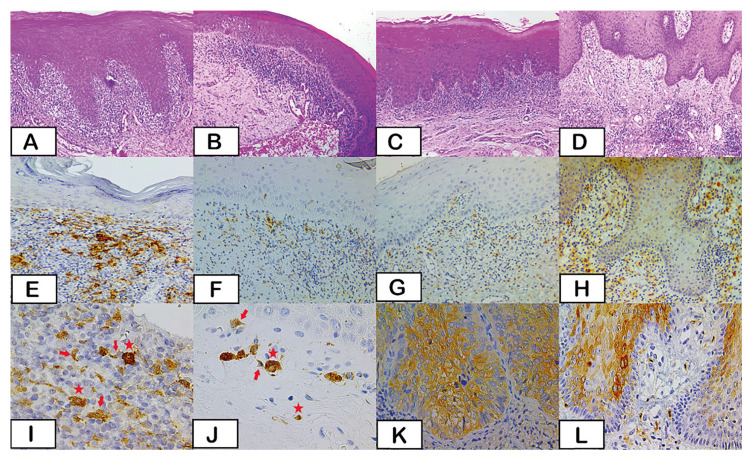
Histopathological and immunohistochemical aspects of lesions. Oral Lichen Planus (A), Oral Lichenoid Lesion (B), Oral Leukoplakia (C), and Unspecific Chronic Inflammation (D) (Hematoxylin and Eosin -H&E- stain 100x). Oral Lichen Planus (E), Oral Lichenoid Lesion (F), and Oral Leukoplakia (G) show negative immunostaining in the epithelium and positive cells in the lamina propria. Unspecific Chronic Inflammation (H) showing ALDH1+ cells in the epithelium and the lamina propria (Polymer-HRP 200x). Details of ALDH1 immunostaining evidencing stellated cells with small vacuums in the cytoplasm (red arrow) and round cells (red star) in lamina propria of Oral Lichen Planus (I) and Oral Lichenoid Lesion (J) and Epithelium of Oral Leukoplakia (K) and Unspecific Chronic Inflammation (L) with confluent and isolated cells (Polymer HRP 600x).

A total of 633 lesions diagnosed as OLP, OLL OLK and UCI were retrieved from histopathological records from 2011 to 2021. After applying the clinical and histopathological eligibility criteria, were selected 16 lesions each group of diagnose, totalizing 64 cases (Fig. 1). Demographic (sex, age), smoking habit and clinical (fundamental lesion and anatomical location) characteristics are shown in Table 2.

- Morphological analysis

The morphological analysis of the ALDH1+ cells is shown in Table 3.

The epithelial expression of ALDH1 was uncommon, with only 1 (6,25%), 2 (12,4%), and 5 (31,2%) cases from OLP, LKO, and UCI, respectively, showing ALDH1+ cells. The epithelium of all LLO lesions was negative for ALDH1 (Fig. 1).

Contrarily, ALDH1+ cells were detected in the lamina propria in all groups of lesions, dispersed along the subepithelial inflammatory infiltrate. All cases from the OLP group and almost all from OLL and OLK (12 and 13, respectively) were ALDH1+ in the lamina propria. Some UCI cases also showed ALDH1-positive cells in lamina propria (Fig. 1). Most of these cells showed a stellated morphology with an angular or spindle-shaped cell body and cytoplasmatic extensions. Some cells also showed amounts of small vacuoles in the cytoplasm. Round or oval cells positive for ALDH1 were also observed (Fig. 1). These were leukocyte-like cells with round nuclei and granular cytoplasm. Isolated cells in the deeper inflammatory infiltrate were sporadic. Positive epithelial cells, when present, were confluent, following the epithelial architecture, or isolated cells (Fig. 1).

- Semiquantitative analysis

**Figure 2:**
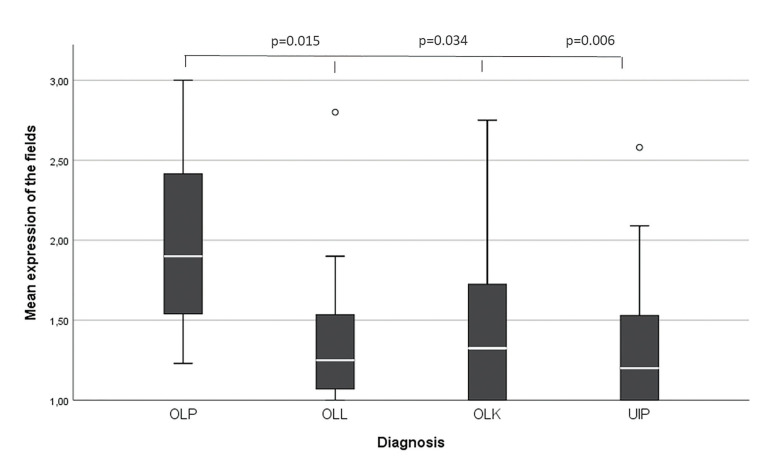
Median expression of ALDH1 in stromal cells of OLL, OLK and UCI in relation to OLP.

The median expression of ALDH1+ cells in the lamina propria was higher for OLP [1.9], followed by OLK [1.3], OLL [1.2] and UCI [1.1] (*p*<0.05), as shown in Fig. 2.

**Table 2:**
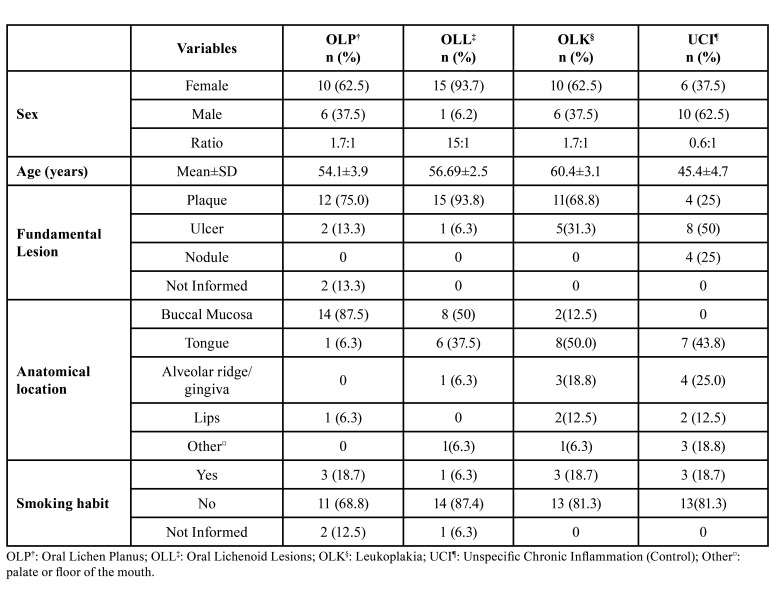
Clinical and demographical data of the 64 included cases.

**Table 3:**
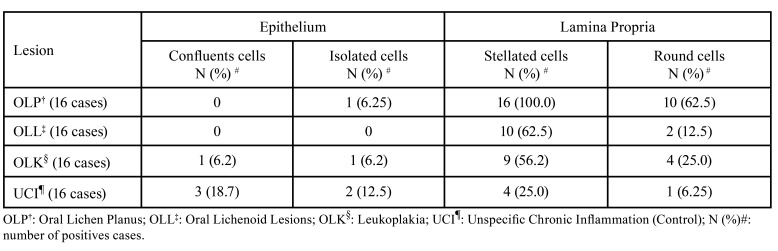
Morphological analysis of ALDH1+ cells in Epithelium and Lamina Propria.

## Discussion

This study aimed to investigate the ALDH1 immunoexpression in a group of lesions presenting a lichenoid inflammatory infiltrate, considering the malignant potential of those lesions (1), and ALDH1 as a marker of cancer stem cells. Additionally, it was also of interest to investigate the role of ALDH1 in the immune-inflammatory infiltrate of OLP.

Strict criteria should be followed when diagnosing OLP, as it can present overlapping clinical and histopathologic features with Oral Lichenoid Lesion and OLK with a lichenoid component, among other lesions. A proposed diagnosis criteria of OLP exclude any lesion with dysplasia (12,15). The term Oral Lichenoid Lesions is preferred for lesions with a definable underlying etiology, as drug reaction (16). OLK can present a band-like chronic inflammatory infiltrate in the superficial lamina propria, mimicking OLP. As a mild dysplasia can occur also as a result of inflammation, the distinction between lesions is not easy, however, the criteria mentioned above help to distinguish them (12).

The malignant potential of OLP has been explored in several studies with controversial results and variable index of transformation (17). On the other hand, it is recognized that other non-dysplastic lichenoid lesions may undergo malignant transformation (11,18,19). ALDH1 is expressed in malignant epithelial cells and oral dysplasia (20). However, our results showed a low expression of ALDH1 in epithelial cells of all group of lesions, with a slight higher expression in UCI cases. These results, differ from others (21), did not give an indication of malignant potential for the lesions investigated here. Although we had a meticulous criterion to select the lesions, and all OLK cases presented dysplasia, a confirmation of this result requires an investigation of a more numerous samples.

Biomarkers have a broad function, the main one being to neutralize inflammatory agents, contributing to the body's defense, minimizing damage and participating in some cases in tissue repair and regeneration (11,14) The ALDH1 is an isoform of aldehyde dehydrogenase, which is expressed in humans as a cytosolic detoxifying isoenzyme that oxidizes intracellular aldehydes and contributes to the oxidation of retinol to retinoic acid in early stem cell’s differentiation. These properties make ALDH1 a marker of cancer stem cells playing an important role in the biology of tumors (6). However the role of ALDH1 in regulation of immune response throughout the control of metabolism of retinol, has not been explored, in especial in oral diseases. In a similar way, the strong association of ALDH1 with cancer stem cells has making the evaluation of this proteins almost restrict to the epithelial cells. Few studies have explored the expression of ALDH1 in stromal cells (14,22). Our results showed an increased number of positive ALDH1 cells in the subepithelial lichenoid inflammatory infiltrate of all lesions. The higher number of positive cells was found in OLP, with a significative difference with the other lesions.

Interestingly, the stromal ALDH1+ cells showed different phonotypes. Most of these cells showed a stellated morphology with a namely angular or spindle-shaped cell body and cytoplasmatic extensions, some of them also showed amounts of small vacuoles in the cytoplasm. Round or oval leukocyte-like cells with round nucleus and a granular cytoplasm in a small number were also present, and isolated cells in the deeper inflammatory infiltrate were sporadically seen. It was not an objective of this study an immunophenotyping of these stromal cells, however call attention the number of them, especially in OLP lesions.

The presence of these phenotypes to ALDH1+ cells were registered in other studies (14,22), which have been associated with a better prognosis or simply considered as stem cells (23). In the present study, we presume that the positive stromal cells are antigen-presenting cells (APCs), including dendritic cells.

OLP arises from an autoimmune response involving T cells producing cytokines that induce a chronic inflammatory response and keratinocytes cell death (17). Activation of T cells depends on antigen presentation by APCs in the context of MHCII receptors. Dendritic cells, a kind of APC control the strength and quality of T and B cell response and are present in both epithelium and lichenoid infiltrate of OLP (24).

Authors demonstrated the presence of retinol in the cytoplasm of ALDH1 + stromal cells (25). Studies also showed that the metabolism of retinol into retinoic acid in DCs mediated by ALDH1 regulate critical parameters of lymphocyte differentiation (8). Moreover, RA enhances the induction of Treg FOXp3 cells by Dcs (8,9).

Studies have demonstrated elevated numbers of Foxp3+Treg cells in OLP lesions compared to healthy control tissues (26,27). If the ALDH1+ cells detected in inflammatory infiltrate of OLP are dendritic cells controlling the lymphocytic differentiation throughout ALDH1, this finding might be a key to understanding the complex immune balance in OLP and an indicative for development of new therapies.

Although this significative suggestion, the present study has several limitations, and the first is the absence of characterization of ALDH1+ stromal cells. However, it is not probable that the difference among the lesions was occasional, and we believe that is associated with the different role of ALDH1+ cells in the unspecific or immune mediated inflammatory infiltrate. New studies are needed to prove our supposition.

## Conclusions

ALDH1 immunoexpression in the epithelium of OLP and lichenoid diseases did not show a contributory tool in the understanding of the potential of malignant transformation of these lesions. However, ALDH1+ cells in lichenoid infiltrate of OLP might indicate a key point in the pathogenesis of this immunoinflammatory disorder.
